# Transinguinal Ultrasound versus Magnetic Resonance in Spica Cast after Closed Reduction of Unstable Hips in DDH

**DOI:** 10.3390/children11030292

**Published:** 2024-02-29

**Authors:** Nicola Guindani, Federico Chiodini, Maurizio De Pellegrin

**Affiliations:** Regional Health Care and Social Agency Papa Giovanni XXIII, 25127 Bergamo, Italy; fchiodini@asst-pg23.it (F.C.);

**Keywords:** van Douveren, transinguinal sonography, developmental dysplasia of the hip (DDH), closed reduction, spica cast

## Abstract

Background. During the treatment of unstable hips in developmental hip dysplasia (DDH), the position of the femoral head must be assessed in spica cast (SC) after reduction. A transinguinal sonographic technique (TIT) to the hip joint has been previously described in the literature. The aim of this study is to evaluate the agreement among TIT and MR to identify hip reduction. Methods. From 2016 to 2019, 14 consecutive newborns (10 female, 4 males) with a mean age of 2.97 ± 1.29 months were treated with closed reduction in SC. A total of 4/14 children had bilateral unstable DDH. Out of 18 hips, there were 8 hips type IV and 10 hips type IIIA, according to Graf. SC were changed monthly and hips were checked both with TIT and MR, looking for persistent dislocation. Results. Overall, a mean of 2.61 SC/hip (mode = 3) was accomplished, accounting for 47 procedures, with 46 reduced hips and 1 dislocated hip: TIT and MR always agreed on the same result (47/47; Cohen k = 1, CI95 1.00 to 1.00). Conclusions. The inguinal ultrasound technique described by van Douveren showed perfect agreement with MR and might be considered a reliable alternative to check the position of the femoral head during the conservative treatment of hip dysplasia in spica cast.

## 1. Introduction

In the conservative management of developmental dysplasia of the hip (DDH) in new-borns, unstable or dislocated hips need to first be reduced, then the femoral head is maintained in the socket with an harness or spica cast, allowing the maturation of the acetabulum [1]. Closed reduction and retention with a pelvi-podalic spica cast (SC) in a “human position”, as described by Fettweis [1,2,3,4], represents a possible treatment option.

**Figure 1:**
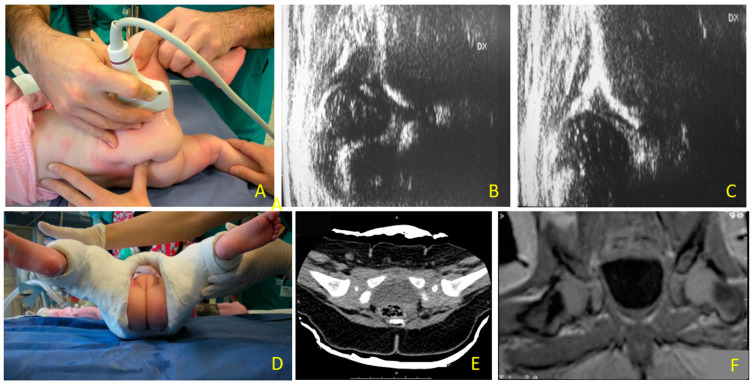
Hip dislocation: (**A**) ultrasound evaluation with lateral approach to the hip joint according to Graf technique during reduction maneuver; (**B**) type IV hip; (**C**) femoral head in the acetabular fossa after closed reduction, before performing a pelvi-podalic spica cast (SC); (**D**) the SC has been applied. With SC, Graf’s technique cannot be applied anymore, to check if the hip is reduced or not, (**E**) to verify the position of the femoral head in SC, computer tomography (CT) or (**F**) magnetic resonance (MR) can be used.

Once SC has been applied, the position of the head must be evaluated, to check if the hip is reduced or not [5]. Different techniques are available to verify the position of the femoral head in SC: X-ray [6], computer tomography (CT) [7,8,9], magnetic resonance (MR) [5,6,10,11] and ultrasound (US) [10,12]. According to Graf’s technique, ultrasound examination should be performed in a frontal standard plane, approaching the hip laterally and avoiding tilting errors of the probe [13] (Figure 1); however, Graf’s method is not applicable in the presence of a SC. More recently, different sonographic techniques have been described, to overcome the problem of hips being covered by a cast [14,15,16]. Among those different US approaches, one of the most used is the transinguinal approach (TIT) described in 2003 by van Douveren et al. [12]. Over the following 20 years, more authors compared TIT with other available techniques as gold standard, such as radiographs or CT [10,17]. 

The aim of this study is to evaluate the agreement between TIT and MR in identifying femoral head position in SC after closed reduction of unstable hips in DDH.

## 2. Materials and Methods

From 2016 to 2019, 14 consecutive patients (18 hips) with DDH and unstable hips were conservatively treated with closed reduction SC according to Fettweis. 

The study was conducted in accordance with the Declaration of Helsinki and approved by the Institutional Review Ethics Committee of Bergamo (ITA), Nr 2021–0134. Informed consent was obtained from patients’ parents/guardians. Included patients were children with DDH and dislocated or unstable hip(s) (Graf type III or IV), treated conservatively with closed reduction in SC. Before every cast changing, each hip was classified according to Graf and a new cast was performed if at least one hip was still dislocated or unstable (Graf type D, III and IV) [10]. If the hip was still dysplastic but not unstable (Graf type IIb or IIc), no further SC was used, and the treatment followed with a brace until the maturation of the acetabulum (Graf type 1). Once maturation was reached, according to the ultrasound criteria described by Graf [1], a check with a single antero-posterior pelvic radiography without gonad shielding was performed [18,19], in order to measure the acetabular index according to Tönnis (AI). If AI showed normal values, then therapy with the brace was interrupted and the patient was scheduled for a long-term follow-up until skeletal maturity, according to Kubo et al. [20,21,22,23].

Each patient was first evaluated as an outpatient, clinically and with ultrasound (both Graf and TIT being performed); only if the hip resulted unstable or dislocated but reducible, a spica cast in narcosis was scheduled in the following days. Before each cast, the patient was re-evaluated in narcosis in the same fashion: clinically and with ultrasound (both Graf and TIT being performed). During the procedure, the position of the hip was checked with TIT and re-checked with TIT immediately after the cast dried, with the patient still in narcosis. This latter TIT after each cast is the one used for the comparison with MR in the present study. TIT was always performed by the same Author (GN).

**Figure 2:**
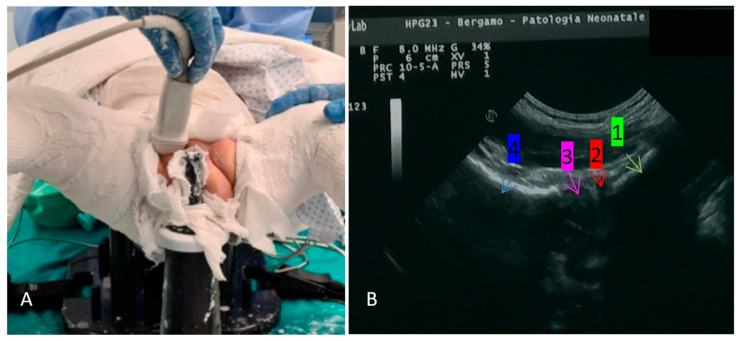
(**A**) The patient is in narcosis on a pediatric table, the SC has already been performed (in human position, according to Fettweis); the right hip is checked with TIT, through the same perineal opening of the cast used for wipers; and (**B**) the sonographic image obtained in the same patient after reduction: 1 ramus superior os pubis, 2 labrum, 3 femoral head, 4 femoral neck.

**Figure 3:**
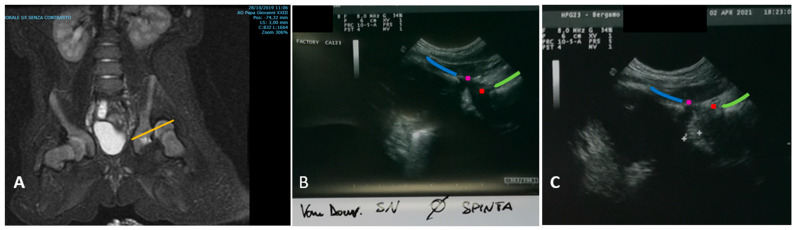
(**A**) MR image (coronal plane) of a dislocated hip (patient Id Nr 4, Graf type IV, see text). The yellow line indicates the plane in which the hips are scanned with the miniconvex probe, using TIT technique. Looking at this image, it is easier to understand why the anatomical landmarks indicated in Figure 2 (ramus superior os pubis, labrum, femoral head and neck) must all be present in the ultrasound images obtained with TIT. Particularly, if the labrum is missing, the femoral head could be dislocated proximally and the iliac wing wrongly identified as os pubis, obtaining a false negative. (**B**) Another patient with a dislocated hip (Graf type IV) examined with TIT. The anatomical landmarks are indicated with the same colors as Figure 2B. The anterior contour of the ramus superior os pubis is marked with a blue line, the anterior border of the labrum is marked with a violet point. The anterior border of the head is marked with a red point and the anterior contour of the neck of the femur is marked with a green line. Here, the hip is dislocated, and the imaginary extension of the lines built by the anterior margin of pubis, labrum, femoral head and neck lies on different curves. (**C**) The same hip from (**B**), after reduction. The hip is now reduced, and the imaginary extension of the lines built by the anterior margin of pubis, labrum, femoral head and neck lies on the same curve.

Each cast was performed in narcosis and replaced every month; after every new cast the hips were checked both with TIT and MR in narcosis, looking for persistent dislocation (Figure 2). With TIT, a hip is defined as dislocated if the imaginary curved line tangent to the ramus superior of os pubis and the femur neck is interrupted [12] (Figure 3). MR was performed within 48 h of the positioning of the cast, with or without narcosis according to radiologist’s preferences and the condition of the child. Every patient was followed-up until hip maturation (according to both Graf classification and Tönnis’ acetabular index [13,20,24]). 

The images obtained with TIT were contextually evaluated by the same orthopedic surgeon who also performed the examination (GN). With TIT, an Esaote MyLab™30 with a miniconvex (Esaote Convex probe 5-2, radius 13 mm, Genoa, Italy) was used, start settings 12 MHz and depth window of 4 cm. The MR was performed with a 1.5 T Siemens (Munich, Germany), using a fast protocol with T2 sequences with 3 mm thickness in 3 planes, evaluated by a radiologist with more than 5 years of experience with pediatric musculoskeletal imaging.

### Statistical Methods

The hypothesis (H_0_) is that TIT performs like MR in detecting the position of the femoral head, whilst the alternative hypothesis (H_1_) is that TIT is less performing than MR [25]. In the present study, MR is considered the gold standard. To test this hypothesis, we compared the results of the TIT (intervention group) with MR (control group).

Sample size has been calculated according to Walter et al. for interclass correlation [26,27], with the following settings: minimum acceptable reliability: 0.95; expected reliability: 0.85; significance level (α): 0.05 (2-tailed); power (1–β): 90%; and expected dropout rate: 5%. This accounts for a sample size, *n* = 33 and sample size (with 5% dropout), n_drop_ = 35. Agreement has been computed according to Cohen’s kappa [25,27].

## 3. Results

In the present study 14 patients (18 hips) were included and according to Graf’s classification there were initially 8 hips type IV and 10 hips type IIIA [28]. Demographic data of the patients are summarized in Table 1.

There were 9/14 female and 5/14 male patients (F/M = 1.8). The mean age at the first procedure was 2.97 ± 1.29 months. A total of 4/14 children had bilateral unstable hip dysplasia. A mean of 2.61 casts/hip (mode = 3) have been performed. At the end of the therapy with cast(s), there were 1/18 still dislocated (Patient Id Nr = 4; initial Graf type IV), 9/18 IIc, 6/18 IIb and 3/18 Ib according to Graf. Patients with the worst hip classified D or worse followed a treatment with cast, whilst patients with the worst hip classified 2c or 2b followed a treatment with a static brace in a human position until maturation to Graf type I and normal acetabular index (Tönnis) according to age [29,30]. A total of 45/47 MR were performed in narcosis and 2/47 without (asleep patients).

In total, 47 procedures (SC in narcosis, TIT and MR) have been performed and in 100% (47/47 events) TIT and MR agreed on the same result (Cohen k = 1, CI_95_ 1.00 to 1.00), (Table 2). There were 46/47 true negative and 1/47 true positive, no false positive or negative, referring to MR as gold standard.

In 1/14 patient (Patient ID: Nr 4; 1/18 hip involved) the conservative treatment was interrupted after the first SC, due to irreducible persistent dislocation. This was the oldest patient (5.8 months at the beginning of the treatment with SC), for whom diagnosis and treatment were delayed because of a prolonged hospitalization for respiratory impairment in the neonatal intensive care unit. The preoperative evaluation occurred in the intensive care unit with the patient still in sedation for respiratory problems and it seemed reducible at that time. Later, the patient underwent an open reduction.

In 1/14 patient (Patient ID: Nr 1; 1/18 hip involved, male), after the first SC the TIT showed a still-dislocated hip, with the patient still in sedation in the operating room. Only in this event, we repeated the entire procedure (SC removal, reduction and TIT) before MR. After re-performing the reduction and SC, in TIT the hip was reduced, thereafter the patient underwent the MR. This broke the study protocol and was undertaken on an ethical basis, to exploit the same sedation and avoid additional procedures. Nevertheless, the Authors decided to include the data, as the concordance between TIT and MR is not biased.

With the exception of Patient Nr 4, all of the others (46/47, 98%) reached the hip maturity with conservative treatment and are still in long-term follow-up for residual acetabular dysplasia [20,21,22,23,31,32].

In total, 13/14 patients achieved the criteria of “mature hips” with an acetabular index (according to Tönnis) > 30° before the brace was removed. The remaining 1/14 (pt ref Nr 4) followed a surgical procedure after the first SC. According to study design and follow-up, we cannot comment on the evolution of the hip during the long-term follow-up, particularly regarding the acetabular index; up to now, no-one needed further treatment.

## 4. Discussion

After closed reduction of unstable or dislocated hips in DDH, the position of the femoral head in spica cast should always be checked [5]. The rate of persistent dislocation is consistent: in up to 10% of the cases, MRI revealed a persistent hip dislocation after application of Fettweis SC in children with unstable hip joint [5]. X-rays are an option that could be performed while performing the spica cast and are also widely available. However, the following problems are present, decreasing accuracy with X-rays: (1) direct visualization of the femoral head is usually not possible (according to patients age, there might be no ossification center of the femoral head), and (2) a persistent dislocation might be overseen with an antero-posterior view of the pelvis, as the dislocation occurs usually on an antero-posterior direction [10]. CT and MR can be considered gold standard, as both of them allow a direct 3-dimensional visualization of the hip [33,34]. MR has the obvious advantage of avoiding radiation and with dedicated simplified sequences it could be performed without sedation and in less than 3 min [35,36]. It is not only the post-reduction check that can be performed with MR, but the procedure itself can also be MR-guided [37]; however, the availability of an operating room with MR for closed reduction of the hip is still a rare option nowadays. In current practice, CT or MR are usually performed only after the spica cast has been performed and any detected failure requires a repeat of the whole procedure. In other words, with CT or MR, the surgeon usually has no way to check the situation of the hip during the reduction maneuver. In contrast, ultrasound techniques are a bed-side test, allow the visualization of the femoral head during the whole procedure and a post-procedural check in spica cast [10,12,17,38,39].

In the present study, TIT was performed comparing results with MR, examinations were performed in the same cohort within 48 h from the positioning of the cast, with or without narcosis. TIT and MR showed a 100% agreement (according to Cohen’s k [25]) in detecting the position in spica cast of the femoral head after closed reduction.

Our data showed a lower incidence of dislocated hips (true positives) in patients treated with SC in comparison to previous studies [10,12,17]. There are many reasons which might explain these data. First, the mean age of our patients was lower, and a younger hip has a higher probability of being reduced in a closed fashion [6,40,41,42,43]. Indeed the mean patient age was 3 months, whilst van Douveren et al. in their milestone work enrolled 13 patients with a mean age of 15 months [12]; also, Beek et al. retrospectively reviewed 33 patients with a mean age of 14 months [17]. On the contrary, Eberhardt et al. (2009) reported on a younger study population (Mean 6.4 weeks, range 1–30 weeks) and found similar results (2/68 dislocated hips). In the present study, there might be a bias drifting towards less difficult cases. The hips reducibility was tested (clinically and with TIT) before performing the procedure and irreducible hips were not included; moreover, TIT was performed during the execution of the SC and this could have influenced the rate of true positives, similarly as in case ID Nr 1. This feature is unique to TIT, in opposition to MR and CT, which are usually [37] used only after reduction and SC completion.

According to van Douveren et al. [12], a hip is defined as dislocated if the modified Shenton line between the ramus superior of os pubis and the femoral neck is interrupted [33]. To date, there are no data concerning the quantification of this interruption, as TIT has only a dichotomous binary outcome (the femoral head is reduced or not). However, the advantage of a simple binary outcome [35,36] could be confirmed in our study, with 100% agreement to the more accurate MR images.

Although TIT was developed as a technique to distinguish if a hip is dislocated or not, Eberhardt et al. already noticed how TIT might conceive further information. The thickness of the connective soft tissue occupying the fossa acetabuli can be visualized, together with the behavior of this soft tissue during the reduction maneuver (is this soft tissue moved apart from the femoral head? Is it still present in the fossa acetabuli after reduction?) [10,44]. These anatomical issues have not been considered nor analyzed in the present study.

The main advantages of the present study include the following: data from consecutive patients were collected prospectively; the control group was based on MR, which is a gold standard for the evaluation of the infant hip [5]; one surgeon used the same protocol and followed-up every single patient. The main limitations of this study must be acknowledged. First, it was designed to investigate the concordance between MR and TIT, not sensibility and specificity. Thus, their computation would not be trustworthy, according to study design and sample size. Second, this study was not blinded, and TIT possibly influenced surgeon’s decisions. In other words, even if this study is observational, TIT might have inferenced the results. Third, TIT was performed once for each procedure, by the same clinician who also evaluated the images: an intra-/interobserver assessment was not performed.

Concerning further clinical developments, the use of a static brace as an alternative to a Pavlik harness and SC to treat critical hips has already been described [10,20,45]. Utilizing bed-side ultrasound techniques to check the position of the femoral head could immediately provide the information that the clinician needs for the treatment [38] in the operating room and in an outpatient setting. Moreover, a preoperative outpatient evaluation with TIT might help to prepare families and clinicians when a reduction in narcosis is planned [4].

## 5. Conclusions

In the present study MR and ultrasound showed perfect agreement in detecting the position of the femoral head. The inguinal ultrasound technique according to van Douveren can be considered a reliable alternative to check the hip in spica cast for the conservative treatment of severe hip dysplasia in infants.

## Figures and Tables

**Table 1:** Demographic data. All patients were treated conservatively with spica cast (SC) after closed reduction. F: female; M: male; worst and best hip in the same patient according to Graf classification ^1^. Nr cast = number of cast(s) performed for each patient during treatment. Nr hip MR = number of MR performed for each patient for each hip; it represents the number of events/patient (e.g., if a patient had 2 involved hips and underwent 3 spica casts, then the Nr Hip MR = 6).

				Hip Classification According to Graf					
				Initial		After The Last Cast			
Ref.	Age at 1st SC (Months)	Sex	Monolateral (Mono) Bilateral (Bil)	Worst Hip	Best Hip	Worst Hip	Best Hip	Nr Cast	Nr Hip MR
1	3.4	F	mono	4	2a	2c	1b	3	3
2	2.5	F	mono	3	1	2b	1b	3	3
3	1.8	M	mono	3	2a	2c	1b	2	2
4	5.8	F	mono	4	2a	4	1b	1	1
5	4.1	F	mono	3	1	2b	1b	3	3
6	2.3	M	bil	3	3	2c	2b	3	6
7	2.7	F	mono	3	1	2c	1b	2	2
8	2.8	M	bil	4	3	2b	2c	3	6
9	3.9	F	mono	3	2b	2c	1b	2	2
10	4.5	F	mono	3	1	2c	1b	3	3
11	1.6	F	bil	3	3	2b	1b	3	6
12	1.1	M	bil	4	3	2c	1b	3	6
13	1.8	M	mono	3	2a	2b	1b	2	2
14	3.3	F	mono	3	1	2c	1b	2	2

^1^ The Graf classification is presented with Arabic number for an easier computation; however, according to Graf it should be in Romanic numbers (4: type IV, 3: type III, 2: type II, 1: type I).

**Table 2:** Comparison between transinguinal ultrasound (TIT) and magnetic resonance (MR) images in spica cast (SC) after closed reduction. TIT and MR results are represented as follows: 1: hip reduced; 0: hip not reduced; * = in the best hip; ** = in the worst hip, according to Graf classification ^1^.

Patient	Pathological Hip(s): Initial Graf	Non-Pathological Hip: Initial Graf	Nr Incl Hip(s)	Nr Cast(s)/Hip	Event(s)/Pt	Prog. Nr of Cast/Hip	Hip Type before SC	TIT	MR	Hip Type after Closed Reduction and SC
1	4	2a	1	3	3	1	4	1	1	3
						2	3	1	1	3
						3	3	1	1	2c
2	3	1	1	3	3	1	3	1	1	D
						2	D	1	1	D
						3	D	1	1	2b
3	3	2a	1	2	2	1	3	1	1	3
						2	3	1	1	2c
4	4	2a	1	1	1	1	4	0	0	4
5	3	1	1	3	3	1	3	1	1	3
						2	3	1	1	D
						3	D	1	1	2b
6	3	/	2	3	6	1 *	3	1 *	1 *	3
						2 *	3	1 *	1 *	D
						3 *	D	1 *	1 *	2c
	3			3		1 **	3	1 **	1 **	D
						2 **	D	1 **	1 **	D
						3 **	2c	1 **	1 **	2b
7	3	1	1	2	2	1	3	1	1	3
						2	3	1	1	2c
8	4	/	2	3	6	1 *	4	1 *	1 *	D
						2 *	D	1 *	1 *	D
						3 *	D	1 *	1 *	2b
	3			3		1 **	3	1 **	1 **	3
						2 **	3	1 **	1 **	D
						3 **	D	1 **	1 **	2c
9	3	2b	1	2	2	1	3	1	1	D
						2	D	1	1	2c
10	3	1	1	3	3	1	3	1	1	3
						2	3	1	1	3
						3	3	1	1	2c
11	3	/	2	3	6	1 *	3	1 *	1*	3
						2 *	3	1**	1 *	D
						3 *	D	1 *	1 *	2b
	3			3		1 **	3	1 **	1 *	D
						2 **	D	1 **	1 **	D
						3 **	D	1 **	1 **	1b
12	4	/	2	3	6	1 *	4	1 *	1 *	3
						2 *	3	1 *	1 *	3
						3 *	3	1 *	1 *	2c
	3			3		1 **	3	1 **	1 **	D
						2 **	D	1 **	1 **	2c
						3 **	2c	1 **	1 **	1b
13	3	2a	1	2	2	1	3	1	1	D
						2	D	1	1	2b
14	3	1	1	2	2	1	3	1	1	3
						2	3	1	1	2c

^1^ The Graf classification is presented with Arabic number for an easier computation; however, according to Graf it should be in Romanic numbers (4: type IV, 3: type III, 2: type II, 1: type I).

## Data Availability

Raw data are already contained within the study. All images presented in this study are not publicly available due to privacy and ethics, but anonymous copies are available on reasonable request from the corresponding author (N.G.).
